# Novel Polymeric Micelles-Coated Magnetic Nanoparticles for In Vivo Bioimaging of Liver: Toxicological Profile and Contrast Enhancement

**DOI:** 10.3390/ma13122722

**Published:** 2020-06-15

**Authors:** Ioana Mihaela Popescu Din, Mihaela Balas, Anca Hermenean, Luce Vander Elst, Sophie Laurent, Carmen Burtea, Ludmila Otilia Cinteza, Anca Dinischiotu

**Affiliations:** 1 Department of Biochemistry and Molecular Biology, Faculty of Biology, University of Bucharest, 91–95 Splaiul Independentei, 050095 Bucharest, Romania; mihaela.ioana.popescu@gmail.com (I.M.P.D.); anca.dinischiotu@bio.unibuc.ro (A.D.); 2 Department of Experimental and Applied Biology, Institute of Life Sciences, Vasile Goldis Western University of Arad, 86 Rebreanu, 310414 Arad, Romania; anca.hermenean@gmail.com; 3 Department of Histology, Faculty of Medicine, Vasile Goldis Western University of Arad, 1 Feleacului street, 310396 Arad, Romania; 4 Department of General, Organic and Biomedical Chemistry, NMR and Molecular Imaging Laboratory, Faculty of Medicine and Pharmacy, University of Mons, 19, Avenue Maistriau, Mendeleev Building, B-7000 Mons, Belgium; luce.vanderelst@umons.ac.be (L.V.E.); sophie.laurent@umons.ac.be (S.L.); Carmen.Burtea@umons.ac.be (C.B.); 5 Department of Physical Chemistry, Faculty of Chemistry, University of Bucharest, 4-12 Regina Elisabeta Blvd, 030018 Bucharest, Romania; ocinteza@gw-chimie.math.unibuc.ro

**Keywords:** polymeric micelles, magnetite nanoparticles, contrast agents, toxicity, MRI

## Abstract

Magnetic nanoparticles are intensively studied for magnetic resonance imaging (MRI) as contrast agents but yet there remained some gaps regarding their toxicity potential and clinical implications of their biodistribution in organs. This study presents the effects induced by magnetite nanoparticles encapsulated in polymeric micelles (MNP-DSPE-PEG) on biochemical markers, metabolic functions, and MRI signal in CD1 mice liver. Three groups of animals, one control and the other ones injected with a suspension of five, respectively, 15 mg Fe/kg bw nanoparticles, were monitored up to 14 days. The results indicated the presence of MNP-DSPE-PEG in the liver in the first two days of the experiment. The most significant biochemical changes also occurred in the first 3 days after exposure when the most severe histological changes were observed. The change of the MRI signal intensity on the T2-weighted images and increased transverse relaxation rates R_2_ in the liver were observed after the first minutes from the nanoparticle administration. The study shows that the alterations of biomarkers level resulting from exposure to MNP-DSPE-PEG are restored in time in mice liver. This was associated with a significant contrast on T2-weighted images and made us conclude that these nanoparticles might be potential candidates for use as a contrast agent in liver medical imaging.

## 1. Introduction

Inorganic nanoparticles including iron oxide ones have been used for a plethora of biomedical applications including cancer theranostic, tissue engineering, regenerative medicine, biosensing, bioimaging, magnetic hyperthermia, and drug delivery systems [1,2,3,4]. Iron oxide nanoparticles can possess different chemical compositions, such as magnetite, maghemite, and mixed ferrites with different magnetic properties [5]. At the nanometer scale, a competition between thermal energy and magnetocrystalline anisotropy occurs in their case, resulting in a transition from the ferromagnetic to a superparamagnetic state, characterized by a high magnetic moment and a low value of coercive field (Hc) which is important to avoid the magnetic particle aggregation in the body. Due to this fact, supermagnetic nanoparticles raise proton relaxation mainly via an outer-sphere mechanism acting as negative (T_2_) contrast agents. In contrast, in the case of positive (T_1_) contrast agents, the inner-sphere relaxation mechanism works [6]. However, the properties of magnetic nanoparticles (MNP) may differ due to the variability from batch to batch. The number of atoms within a cluster and the occurrence of defects is beyond control determining that not all nanoparticles bear a sizable magnetic moment. The properties of nanoparticles can also influence the cellular responses to their toxicity [7,8]. Recently, it was proved that magnetite (Fe_3_O_4_) nanoparticles were better tolerated by PC12 cells, compared to maghemite ones (that induced significantly programmed cell death), due to their catalase-like activity that could counteract the reactive oxygen species induced postexposure [9].

Nude iron oxide nanoparticles exhibit relatively high toxicity and coatings are mandatory to enhance their biocompatibility, improve the internalization in many cell types, and also distribution, cytotoxicity, and clearance [10]. However, the ability of the MNP to perform efficiently as diagnostic tools is closely related to the chemical structure, reactivity, and biocompatibility of the materials used to cover their surface [11]. So far, different materials have been used for the coating of MNP, such as: organic molecules and surfactants [12,13], natural and synthetic polymers such as polyethyleneimine [14], polyethylene glycol (PEG) [15,16], polyvinylalcohol, polyvinylpyrrolidone [17], polyacrylic acid, poly (N-isopropylacrylamide), dextran [18,19], chitosan [20,21], pullulan, gelatin [22,23], lipids, proteins, in addition to some metals such as gold [24], silver, and nonmetal and metal oxides such as silicon oxides, aluminum oxides, etc. [25].

Previous in vivo studies have shown that PEG-coated MNP exhibits excellent stability in different physiological solutions, while lipid-carrier polymeric micelles confer an adequate hydrophobicity to MNP that contributed to the maintaining of their biocompatibility [26,27]. Polymeric micelles are nanoscopic aggregates usually composed of amphiphilic block copolymers [28,29,30]. The micellar core is hydrophobic and may serve as a natural carrier for hydrophobic drugs, contrast agents, or both in one nanoparticle [31,32,33]. PEG is a polymer with solubility in aqueous solutions, very low toxicity, immunogenicity, antigenicity, and high flexibility of its polymer chain, thus, the PEG-ylated derivatives are usually used in functionalization of nanoparticles to increase the biocompatibility [16,34].

The polymeric coating confers many characteristics to the nanoparticles required for their uses in biomedical applications. For example, it promotes avoiding opsonization [35,36], prevents their agglomeration [37], and impedes the accumulation of nanoparticles in cells of the reticuloendothelial system [38]. The polymeric structures could also limit the contact of iron oxides with the intracellular structures, preventing the formation of free radicals through Fenton and Haber Weiss reactions. Previously, it has been shown that dextran [39] and PEG [40] coatings successfully fulfilled this role. 

Although more and more studies (both in vitro and in vivo) have been conducted on the toxicity caused by exposure to different types of contrast agents, the exact mechanism of action and elimination pathway from the body is not yet known.

One of the main routes of MNP distribution in the human body starts with their uptake by Kupffer cells [41] and macrophages resident in the red pulp of the spleen. Then, MNPs are digested by the lysosomes of Kupffer cells and macrophages and once iron is released, it is restored in the body and follows the normal metabolic pathways of elimination, i.e., renal clearance, being incorporated into hemoglobin [42] or bound to different proteins with chelating properties such as ferritin and transferrin [43].

An increased level of free cellular iron in the body is toxic and may result in the induction of oxidative stress [44]. For example, iron ions (Fe^2+^ and Fe^3+^) that are formed by MNP metabolism can generate reactive oxygen species (ROS) through the Fenton or Haber Weiss reactions [45,46,47]. A high level of ROS activates the enzymatic and nonenzymatic antioxidant systems. MNP exposure can enhance ROS production through prooxidant functional groups on the reactive surface of MNP and redox cycle activation [47]. When the cellular antioxidant systems fail to counteract the excess of ROS, oxidative stress is installed and oxidations of biomolecules occur. 

Prooxidant metals such as iron induce lipid peroxidation, one of the end products of this process being malondialdehyde (MDA) [48]. The uncontrolled generation of ROS triggers also a cascade of pro-inflammatory cytokines and mediators by activating MAPK (mitogen-activated protein kinase) signaling pathways and the nuclear factor NF-kB (nuclear factor-kappa B) [49,50], which controls gene transcription for pro-inflammatory cytokines such as IL-1β, IL-8, and TNF-α, involved in the pathogenesis of fibrosis [47].

This study was conducted to investigate the potential and performance of MNP coated with a modified phospholipid micelle layer as contrast agents for liver MRI applications. The effects induced on some serum biomarkers and oxidative stress ones in hepatic tissue were also analyzed.

## 2. Materials and Methods

### 2.1. Nanoparticles

The preparation and characterization of polymeric micelles loaded with MNP used in this study were previously described [51]. The hydrophobic magnetite (Fe_3_O_4_) nanoparticles were synthesized using a solvothermal method with minor modifications [52] using oleic acid as a capping agent. Thus, to become water-dispersible, MNP were encapsulated in the core of polymeric micelles of 1,2-Distearoyl-sn-glycero-3-phosphoethanolamine-N-(methoxy (poly(ethylene glycol))-2000) (ammonium salt; DSPE-PEG, Avanti Polar Lipids, Alabaster, AL, USA). The preparation of the polymeric micelles was performed using the dry film hydration procedure. Briefly, PEG-ylated phospholipid derivative was dissolved in a minimum volume of chloroform and the lipid was deposited as a thin film on the walls of a round bottom flask, under reduced pressure. After the total removal of the organic solvent, the lipid film was rehydrated with saline solution to a concentration of 20 mg/mL PEG-DSPE. Micelles encapsulating magnetic material were obtained by mixing the MNP with lipids during the film preparation. Finally, the micelle dispersion was filtered through a Millex filter (0.22 µm diameter, Merck KGaA, Darmstadt, Germany) to remove possible aggregates.

The MNP obtained were nearly monodispersed with a polydispersity index 0.086 obtained with a Zetasizer Nano-ZS90 Malvern analyzer (Malvern Instruments Ltd., Malvern, UK), and an average size of 12.5 nm. The average size of the coated MNP was 21.5 nm, slightly higher than the size of unloaded (empty) micelles prepared from phospholipid polymeric derivative DSPE-PEG. The surface potential of the unloaded and loaded DSPE-PEG micelles appeared similar [51]. The dispersion was stable, without any visible appearance of solid magnetic precipitate or variation of the micelle size for up to three months. The DSPE-PEG micelles loaded with MNP were used in the experiment within two weeks of preparation from the same batch.

### 2.2. Animals

The present study was performed on CD1 mice, of both sexes, weighing 25–30 g from the Animal Facility of "Vasile Goldiș" Western University of Arad and the NMR and Molecular Imaging Laboratory from the University of Mons (Place du Parc 20 7000 Mons, Belgium) from Mons, Belgium. The animals were hosted in IVC cages under ad libitum feeding and watering conditions, with a controlled environment (temperature, humidity, and lighting). Experimental procedures were done according to Directive 2010/63/EU and national legislation (Law No. 43/2014). The experimental protocol was approved by the Vasile Goldis Western University Ethics Committee.

### 2.3. Experimental Design

A total of 105 mice were randomly selected and divided into five groups corresponding to five exposure intervals: 1, 2, 3, 7, and 14 days. These groups were divided into three groups of seven individuals each. The administration of MNP encapsulated in DSPE-PEG micelles (MNP-DSPE-PEG) was performed by intravenous injection (in the tail vein) of mice at different concentrations, as follows: 5 control groups were injected with 0.7% sodium chloride solution without nanoparticle suspension (n = 7 mice/group), and the other 10 groups with two different concentrations of the nanoparticle suspension prepared in sodium chloride 0.7%; 5 lots with a dose of 5 mg Fe/kg body weight (bw) and the other 5 groups with the dose of 15 mg Fe/kg bw (n = 7 mice/group per dose). After mice euthanasia, the liver tissue was collected according to the five predetermined time intervals. Biological samples were divided into two tissue fragments to be processed properly up to the moment of biochemical (cryopreservation at a temperature of −80 °C) and histopathological (paraformaldehyde fixation and paraffin embedding) determinations.

### 2.4. Biochemical Analysis

The serum activities of aspartate aminotransferase (AST), alanine aminotransferase (ALT) and gamma-glutamyl transpeptidase (GGT) were evaluated by the spectrophotometric method using commercially available kits (Roche reagents, Meylan, France) according to the manufacturer’s instructions.

### 2.5. Histology

The liver biopsies were fixed in Bouin solution, embedded in paraffin, sectioned at 5 μm and stained with hematoxylin and eosin (H&E). The Prussian Blue/Perls staining was performed using a kit from Titolchimica (Veneto, Italy), to detect the presence of iron accumulation in the tissues exposed to MNP-DSPE-PEG. The slides were deparaffinized, washed, and immersed for 20 min in a mixture of potassium ferrocyanide solution and, respectively, activation acidic buffer, followed by incubation with Mayer’s carmalum according to the kit instructions. The microscopic sections were analyzed under the microscope (Olympus BX43 (Olympus, Tokyo, Japan) equipped with an Olympus XC30 digital camera and CellSens software, v1.11, Olympus). The reactive ferric iron appeared colored in blue to green and the nuclei appeared colored in purple-red.

### 2.6. Nuclear Magnetic Relaxation Dispersion (NMRD) Profile and Relaxivity Measurements

To determine the magnetic character of the MNP-DSPE-PEG, the ^1^H NMRD profile was acquired on a Fast Field Cycling NMR relaxometer (Stelar, Mede, Italy), which measures the evolution of the longitudinal relaxation time of water protons (T_1_) as a function of proton Larmor frequency expressed in MHz. NMRD profiles of contrast agents provide at a glance their relaxation efficiency (or relaxivity) at different Larmor frequencies, which is valuable information for MRI applications at a given field.

The efficiency of a MR contrast agent is expressed by its relaxivity (r_1_ and r_2_), which is defined by the longitudinal (R_1_ (s^−1^) = 1/T_1_) and transverse (R_2_ (s^−1^) = 1/T_2_) relaxation rates observed for a 1 mM aqueous solution of contrast media, of which the R_1_ or R_2_ of pure water (diamagnetic R_1,2_^dia^) is subtracted (r_1_ = (R_1_ − R_1_^dia^)/C and r_2_ = (R_2_ − R_1,2_^dia^)/C) [53]. The r_1_ and r_2_ values are reported in s^−1^ mM^−1^ and are field and temperature-dependent. 

The measurement of T_1_ and T_2_ relaxation times of the nanoparticle suspension (1 mM Fe) was performed at 37 °C using two Bruker Minispec analyzers (Bruker, Karlsruhe, Germany), one working at 60 MHz (1.4T, mq-60) and the other one at 20 MHz (0.47T, mq-20).

### 2.7. Biodistribution of MNP-DSPE-PEG in Mice Liver Evaluated by Relaxometry

The biodistribution of MNP-DSPE-PEG in liver tissue was evaluated by measuring the transverse (T_2_) relaxation times. In this regard, CD1 mice of both sexes were injected into the lateral tail veins with two concentrations of the MNP-DSPE-PEG suspension (6 mice with 100 μmol Fe/kg bw and 6 mice with 200 μmol Fe/kg bw). Three mice for each dose were sacrificed after 1 and 2 days and the organs were collected to measure the T_2_ at 60 MHz and 37 °C on a Bruker Minispec mq-60 analyzer.

### 2.8. Magnetic Resonance Imaging (MRI) Analysis of Nanoparticle Distribution in the Liver Tissue

Three mice anesthetized with 2% isoflurane (Tem Sega, Lormont, France) in airflow (flow rate of 70 mL/min) were monitored in terms of respiratory rate throughout the experiment. Using a hot water circulation system, the body temperature of the mice was maintained at 36–37 °C. MRI images were acquired at the thoracoabdominal region with a 7T (300 MHz) Bruker Biospec imaging system (Bruker, Ettlingen, Germany), equipped with a horizontal Pharmascan magnet and a circularly polarized volume coil (55 mm × 23 mm; frequency 3 MHz; maximum 5 ms RF), before (precontrast) and after injection of the nanoparticles (postcontrast). The injected dose of nanoparticle suspension was of 100 μmol Fe/kg bw equivalent of ~5 mg Fe/kg bw. The imaging protocol used for image acquisition was RARE (Rapid Acquisition with Relaxation Enhancement) with the following parameters: TR (repetition time) = 3000 ms, RARE factor = 4, TE (echo time) = 48.7 ms, NEX (number of echoes) = 8, matrix = 256 × 256, FOV (field of view) = 3.5 cm, slice thickness 1.50 mm, 20 coronal slices and 20 axial slices, spatial resolution = 137 μm, TA (acquisition time) = 25 min 36 s. 

The contrast enhancement was calculated using the ImageJ software (v. 1.47q, National Institutes of Health, Bethesda, MD, USA), after measuring the signal intensity (SI) values on pre- and postcontrast MR images within regions of interest (ROI) drawn manually at the level of the liver. The standard deviation (SD) of the noise was measured in a region outside of the animal body. The changes in Signal-to-Noise Ratio (SNR) of postcontrast MRI images as compared to precontrast ones were expressed as percentages (ΔSNR%) and quantified as previously described [54].

### 2.9. Preparation of Liver Tissue Total Extract

The total protein extract was obtained from 0.1 g of mouse liver tissue (previously stored at −80 °C) homogenized in 1 mL of 0.1 M Tris/5 mM EDTA buffer, pH 7.4 and sonicated on ice 3 times for 30 s, using an Ultrasonic Processor UP50H from Hielscher (Hielscher Ultrasound Technology, Teltow, Germany) set at 1 cycle and 80% amplitude. After 1 h of incubation at 4 °C, the tissue homogenates were centrifuged at 10,000 rpm and 4 °C for 30 min. In the end, the supernatant was collected for further biochemical determinations. The total protein concentration was measured using Lowry’s method (1951) and bovine serum albumin (BSA, Sigma-Aldrich, St. Louis, MO, USA) as standard [55].

### 2.10. Measurement of Enzymatic Activities

The activities of catalase (CAT), superoxide dismutase (SOD), glutathione peroxidase (GPX) and glutathione reductase (GR) enzymes were measured as previously described [51]. The GST activity was assayed by recording the rate of 1-chloro-2,4-dinitrobenzene (CDNB) conjugation with GSH at 340 nm for 5 min at 25 °C, according to the Habig et al. method [56]. The molar extinction coefficient of CDNB (ɛ_CDNB_ = 9.6 × 10^3^ M^−1^·cm^−1^) was used to calculate the variation of GST activity. The glucose-6-phosphate dehydrogenase (G6PDH) activity was estimated using the method of Beutler which is based on the reduction of NADP+ by G6PDH in the presence of glucose 6-phosphate [57]. The increase in NADPH concentration was monitored for 5 min at 340 nm and 25 °C. The molar extinction coefficient of NADPH (εNADPH = 6.22 × 10^3^ M^−1^·cm^−1^) was used for calculation. All the enzymatic activities were expressed as specific activities (U/mg of protein) and represented as % from controls.

### 2.11. Quantification of Reduced Glutathione (GSH) Concentration

The GSH concentration was determined using the Glutathione Analysis Kit (CS0260, Sigma-Aldrich, St. Louis, MO, USA), according to the manufacturer’s instructions. The total protein extracts were deproteinized with an equal volume (1:1) of 5% sulfosalicylic acid (SSA) and then centrifuged at 10,000 rpm for 30 min and 4 °C. After centrifugation, a volume of 10 μL of each supernatant obtained was pipetted into a 96-well plate (Falcon Tissue Culture Plate, Becton Dickinson Labware) together with 150 μL working mixture (1.5 mg/mL of 5.5’-dithiobis-(2-nitro benzoic acid) prepared in 100 mM potassium phosphate, pH 7.0 and 1 mM EDTA). After 5 min of incubation at room temperature, the absorbance was read at 412 nm using a microplate reader. The results were expressed in nmol/mg protein.

### 2.12. Lipid Peroxidation Assay

The level of lipid peroxides was estimated by fluorimetric analysis of malondialdehyde (MDA), a marker of polyunsaturated fatty acid peroxidation according to the method described by Del Rio et al. [58]. A volume of 200 μL total proteic extract from each sample appropriately diluted was mixed and incubated with 700 μL 0.1 N HCl for 20 min at room temperature. Thereafter, 900 μL of 0.025 M thiobarbituric acid (TBA) was added and a second incubation was performed at 37 °C for 65 min. Subsequently, 400 μL of 0.1 M Tris-HCl buffer/5 mM EDTA, pH 7.4 was added and the fluorescence was measured at λex = 520 nm and λem = 549 nm. The MDA concentration in the samples was estimated using a calibration curve (1 µM MDA stock solution). The results were expressed in nmol/mg protein.

### 2.13. Measurement of Oxidative Modifications of Proteins

a. The protein thiol groups (P-SH) concentration was measured using 4,4′-dithiodipyridine (DTDP) according to the method adapted by Riener et al. [59]. This method is based on the reaction between P-SH and DTDP which results in the formation of 4-pyridyldithio-derivatives (R-S-S-Pyr). A secondary reaction can occur between these R-S-S-Pyr derivatives and other P-SH groups with the formation of symmetrical products with disulfide bridges (R-S-S-R). Briefly, a volume of 100 μL of the total protein extract was deproteinized with 100 μL of trichloroacetic acid (TCA) 20%, and the mixture was left on ice for 10 min. The samples thus prepared were centrifuged for 10 min at 10,000 rpm and 4 °C. Then, the supernatant was carefully removed and a volume of 20 μL 1 M NaOH solution was added to the remaining pellet. After complete dissolution of the pellet, 730 μL 0.4 M Tris buffer, pH 9.0, and 30 μL 4 mM DTDP solution were added. After incubation in the dark for 5 min at room temperature, the absorbance was read at 324 nm. The concentration of P-SH was estimated using a calibration curve with N-acetyl cysteine (in the range of 0–100 µM) and the results were expressed in nmol/mg protein.

b. The advanced oxidation protein products (AOPP) level was measured according to a method adapted by Witko-Sarsat et al. using a 100 mM chloramine-T stock solution as standard [60]. Briefly, a volume of 200 μL diluted total protein extract was mixed with 10 μL KI for 5 min at room temperature in a 96 well plate. Then, 20 μL of glacial acetic acid was added to this mixture and stirred for 30 s at room temperature. Optical density was read at 340 nm. The AOPP concentration was calculated using a calibration curve and expressed in μmol chloramine/L.

### 2.14. Statistical Analyses

Data were analyzed using GraphPad Prism software (version 6, Inc., La Jolla, CA 92037 USA) and expressed as mean values ± standard deviation (SD). The comparison between the working groups was made by the one-way ANOVA test followed by a Bonferroni posthoc test. The statistical significance was set at a probability value (p) less than 0.05.

## 3. Results

### 3.1. Variation of AST, ALT and GGT Enzymatic Activities in Mice Blood Serum

**Figure 1:**
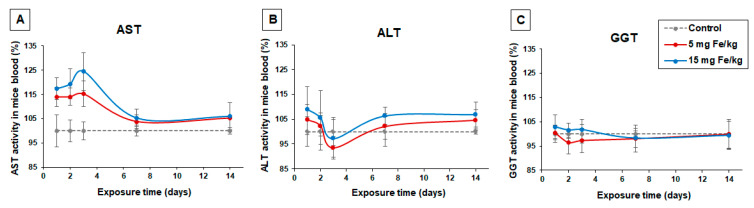
Effect of MNP-DSPE-PEG on the activity of some blood enzymes in the CD1 mice up to 14 d: (**A**) aspartate aminotransferase (AST), (**B**) alanine aminotransferase (ALT) and (**C**) Gamma-glutamyl transpeptidase (GGT). The results were expressed as mean ± standard deviation (SD) and represented as percentages related to the control group.

The enzymatic activities of AST, ALT, and GGT are blood markers for liver injuries. The results showed minor changes in these enzyme activities in mice treated with the two doses of nanoparticles (Figure 1) compared to control. The activity of AST started to increase after first-day postadministration of MNP-DSPE-PEG to CD1 mice and reached a maximum in the third day when the AST activity level was higher by 15% (5 mg Fe/kg bw) and, respectively, 24% (15 mg Fe/kg bw) compared to control. In the same conditions, the ALT activity slightly increased on the first day of the experiment by 4% and, respectively, 9% but then started to decrease getting little below the level of control on the third day. The variation of GGT in this period was almost undetected (Figure 1C). After 7 days of recovery, the activity of all three enzymes was registered.

### 3.2. Iron Biodistribution in Liver Tissue

#### 3.2.1. Histochemistry Studies

**Figure 2:**
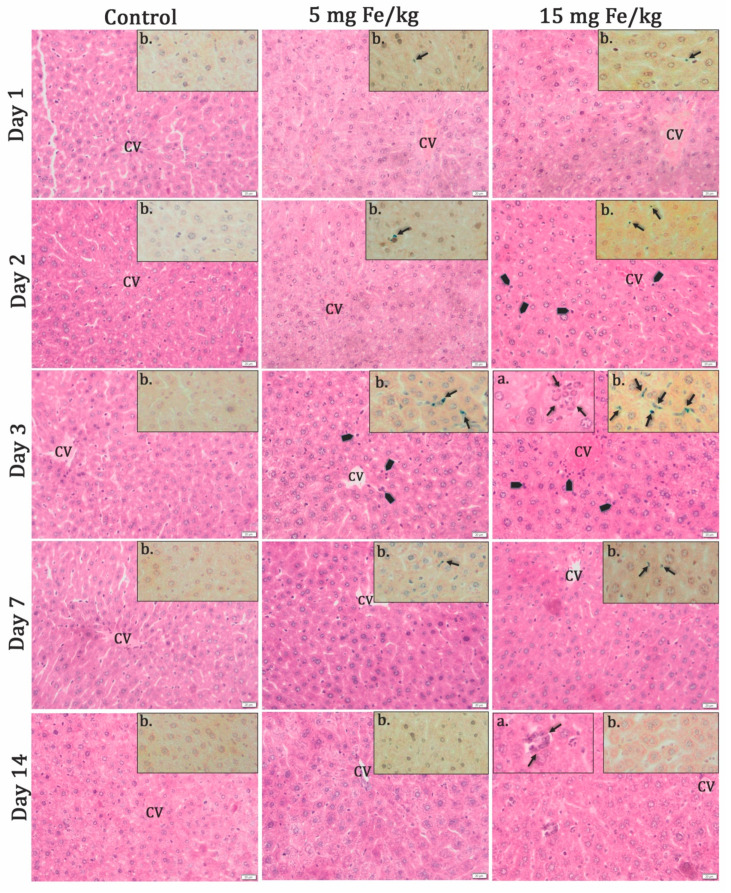
The histopathological aspect of the liver tissues at 1, 2, 3, and 7 d of MNP-DSPE-PEG injection. Hematoxilin&Eosin staining; CV—centrilobular vein; arrowhead—inflammatory cells; (**a**) detail rectangles—granulomas with giant cells; (**b**) detail rectangles—Prussian blue staining of the presence of iron deposits (blue color indicated by arrows) in the pathological tissue slides after 1, 2, 3, and 7 d post-MNP injection. Scale bar: 20 μm.

Histopathological changes occurred after MNP-DSPE-PEG injection and were maximal after 3 d, after which they returned to the normal aspect, comparable to control, after 14 days (Figure 2). After 2 days, activated Kupffer cells are observed, as well as infiltration of inflammatory cells in the liver parenchyma, especially for the high concentration of MNP. At this time interval, granulomas and especially giant cells were noticed (Figure 2a).

Prussian blue staining of the iron deposits in the tissues revealed the presence of MNP in sinusoids, starting with Day 1 and the traffic enhanced further so that, after Day 2, they were highlighted at the level of Kupffer cells (Figure 2b). After 7 d, they were sporadically present and two weeks later left parenchyma, although the granulomas were still observed on the histological sections for high concentration.

#### 3.2.2. Relaxometric Studies

**Figure 3:**
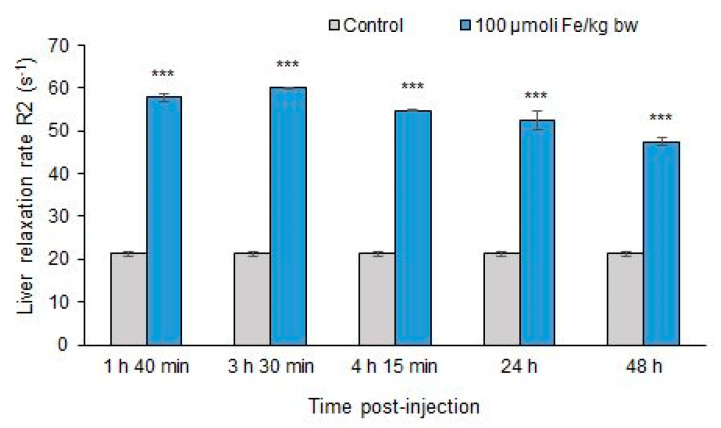
Liver relaxation rate R_2_ (s^−1^) following exposure of mice at 100 μmol Fe/kg bw of MNP- DSPE-PEG up to 48 h. Values are represented as mean ± standard deviation (SD) and considered statistically significant when *** *p* < 0.001 versus the control group.

The biodistribution of MNP-DSPE-PEG in liver tissue has been confirmed by measuring the transverse relaxation rate R_2_ at several time points after injecting 100 µmoles Fe/kg bw (Figure 3). The variation of R_2_ in the liver of mice injected with 100 μmoles Fe/kg bw was monitored at several time points: 1 h 40 min, 3 h 30 min, 4 h 15 min, 24 h, 48 h. The results showed a significant increase of R_2_ in the excised liver from injected mice related to the control, demonstrating the liver uptake of MNP-DSPE-PEG. We also noted that in the first 3 h and 30 min, R_2_ reached its maximum and then gradually began to slightly decrease up to 48 h for the mice liver exposed to a dose of 100 μmoles Fe/kg bw.

### 3.3. Efficacy of MNP-DSPE-PEG as Contrast Agents

**Figure 4:**
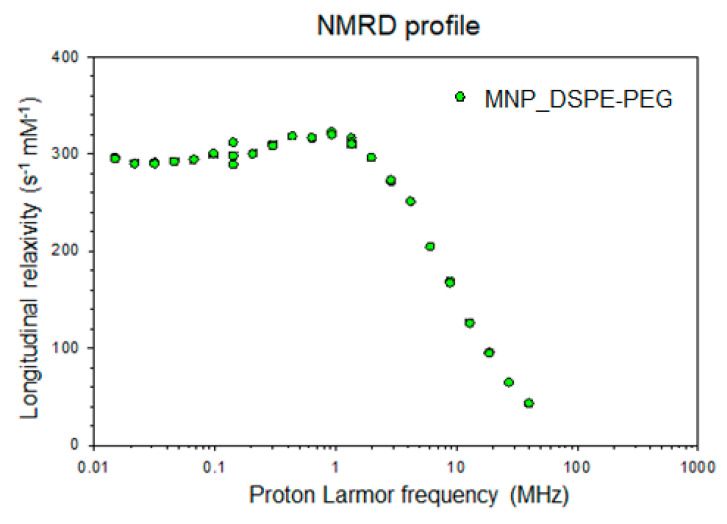
NMRD profile of MNP-DSPE-PEG acquired at 37 °C.

The magnetic character of the MNP-DSPE-PEG was revealed by the NMRD (nuclear magnetic resonance dispersion) profile (Figure 4). Thus, the results showed that MNP encapsulation with the DSPE-PEG micelles shell did not change the magnetic properties of MNP used in this study.

The measured values of relaxivity are presented in Table 1. These data indicate that our MNP are superparamagnetic contrast agents, presenting a higher r_2_/r_1_ ratio at high magnetic fields, being thus susceptible to produce a negative contrast.

**Figure 5:**
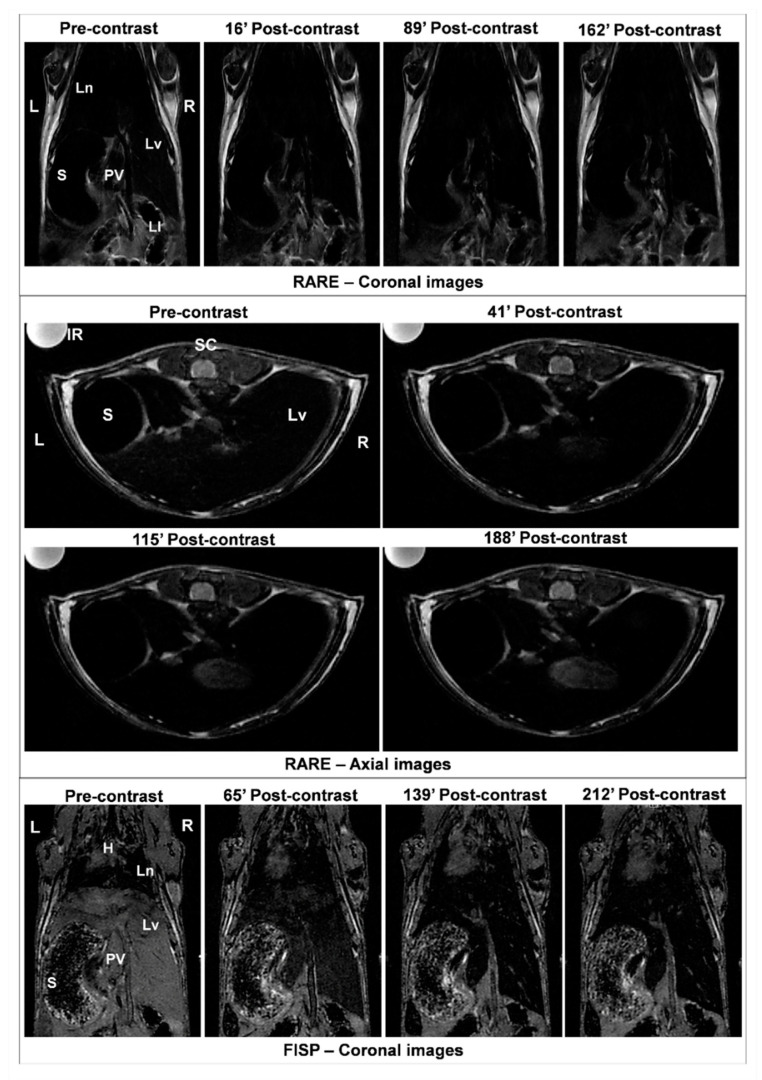
MRI images (coronal and axial RARE, and coronal FISP) of the mouse body acquired with a T_2_-weighted spin-echo sequence before injection (precontrast) and after injection of MNP-DSPE-PEG (postcontrast) at a dose of 100 μmoles Fe/kg bw, at different time intervals. Abbreviations: L: left, R: right, Ln: lungs, S: stomach, PV: portal vein, Lv: liver, LI: large intestine, SC: spinal cord, H: heart.

To validate the potential as contrast agent of MNP-DSPE-PEG, MRI images (Figure 5) were acquired at the thoracoabdominal level of CD1 mice using a T_2_-weighted spin-echo sequence before and after injection of the contrast media at a dose of 100 μmoles Fe/kg bw. Precontrast images were considered as a control for analyzing postcontrast images and changes appeared in signal intensity up to 4 h. The MNP injection induced an obvious negative contrast of the liver in both the coronal and axial sections (Figure 5) indicating the efficacy of MNP-DSPE-PEG as a T_2_ contrast agent for their use in diagnosis. A negative contrast enhancement (ΔSNR%) in the liver was obtained after evaluation of postcontrast RARE axial images as follows: −61.64% after 41 min, −81.08% after 115 min, and −58.74% after 188 min. These results confirm the empirical observation of MR images.

### 3.4. Variation of Enzymatic Activities in Mouse Liver Tissue

**Figure 6:**
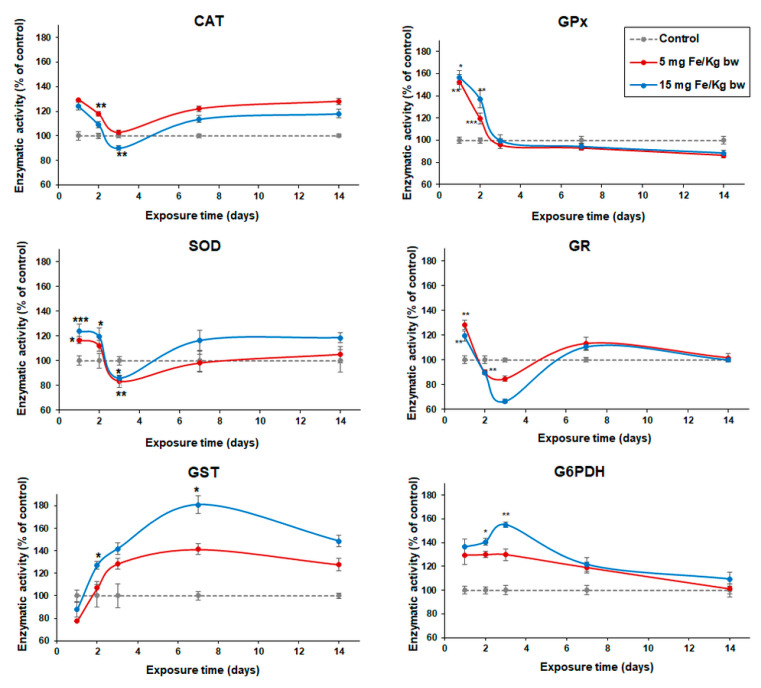
Variation of enzymatic activity of CAT, SOD, GPx, GR, GST, and G6PDH in the liver of CD1 mice treated with 5 and 15 mg Fe/kg bw of MNP-DSPE-PEG after 1, 2, 3, 7, and 14 d from the administration. Values are represented as mean ± standard deviation (SD) and are expressed as a percentage of control; the results were considered statistically significant when * *p* < 0.05; ** *p* < 0.01; *** *p* < 0.001 versus the control group.

After injection of the CD1 mice with MNP-DSPE-PEG, the activity of several enzymes (CAT, SOD, GPx, GR, GST, G6PDH) was monitored up to 14 days (Figure 6). Both SOD and CAT activity followed the same trend but showed different responses between doses. Thus, the CAT activity increased significantly in the first day after treatment, by 29% in mice exposed to 5 mg Fe/kg bw and by 24% in those exposed to 15 mg Fe/kg bw, and then gradually decreased up to 3 days. After one and two weeks, the CAT activity registered an increase in both conditions up to the level of control. Similarly, on the first day, SOD activity recorded the maximum increase compared to control, by 16% in mice exposed to 5 mg Fe/kg bw dose and by 24% in those exposed to 15 mg Fe/kg bw dose. Starting with the second day, the activity of SOD began to drop with 4% for both doses (only by 12% and 20% higher than control) and after three days of exposure, for the same conditions, the SOD activity significantly decreased below the control level by 17% and, respectively, 14%. Following this period, no statistically significant variation was observed for up to 14 d, showing a recovery of the SOD activity. The dose ratio was maintained throughout the entire experimental period. The variation of SOD activity was directly proportional to the dose while the CAT activity was inversely proportional. 

On the other hand, a variation of the GPx activity in the hepatic tissue following exposure to the MNP-DSPE-PEG was observed only in the first two days of the experiment. The results showed a significant increase of the GPx activity by 52% on the first day and by 19% on the second day in mice injected with a dose of 5 mg Fe/kg bw, respectively, by 56% and 36% in mice injected with a dose of 15 mg Fe/kg bw, compared with the activity recorded in the control group. At the other exposure intervals, the GPx activity remained close to that of control. Opposed to GPx activity, the GST activity gradually increased depending on dose starting from the second day of exposure up to 14 d recording a maximum after 7 d by 41% for the low dose (5 mg Fe/kg bw) and by 81% for the high dose (15 mg Fe/kg bw) compared to the control group. At the same time, GR activity significantly increased on the first day of the exposure to MNP-DSPE-PEG in both groups by 28% (5 mg Fe/kg bw) and, respectively, by 19% (15 mg Fe/kg bw) and then decreased. On the third day, it was recorded the maximum decline by 15% (5 mg Fe/kg bw) and, respectively, by 34% (15 mg Fe/kg bw) for the same conditions compared to the control group. At 7 d postexposure, the activity of GR reached the control values.

The variation of G6PDH activity in the liver was dose-dependent. We observed an increase of activity in the first 3 d after injection by 29% (after 1 d), 29% (2 d), and 30% (3 d) in the liver of mice injected with a dose of 5 mg Fe/kg bw and, respectively, by 36%, 40%, and 55% in mice injected with a dose of 15 mg Fe/kg bw compared to control. After this period, the level of G6PDH activity was back to normal (similar to the control group).

### 3.5. Variation of Intracellular GSH Content

**Figure 7:**
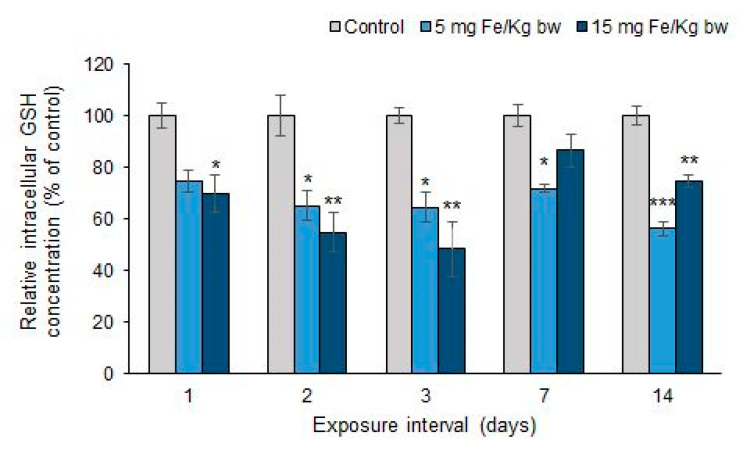
Effect of exposure of CD1 mice at doses of 5 and 15 mg Fe/kg bw of MNP-DSPE-PEG on the level of GSH in the liver, after 1, 2, 3, 7, and 14 d. Values are calculated as mean ± standard deviation (SD) and expressed as percentages related to the control group; the results were considered statistically significant when * *p* < 0.05; ** *p* < 0.01; *** *p* < 0.001 versus the control group.

The injection of MNP-DSPE-PEG in CD1 mice leads to a decrease of the GSH level in hepatic tissue below the GSH concentration found in the control group at all exposure intervals. As can be seen in Figure 7, the lowest level of GSH content was recorded after 3 d of exposure, with a decrease by 36% and, respectively, by 52% for doses of 5 and 15 mg Fe/kg bw, compared to the control. The decrease of GSH level was maintained up to 14 d and interestingly, the level observed in mice injected with the high dose was higher compared with the one found in mice injected with the low dose after 7 d of exposure.

### 3.6. Oxidative Modifications

Oxidative modifications of lipids and proteins were assessed in mice hepatic tissue to estimate the degree of oxidative stress induced by the exposure to the MNP-DSPE-PEG. 

**Figure 8:**
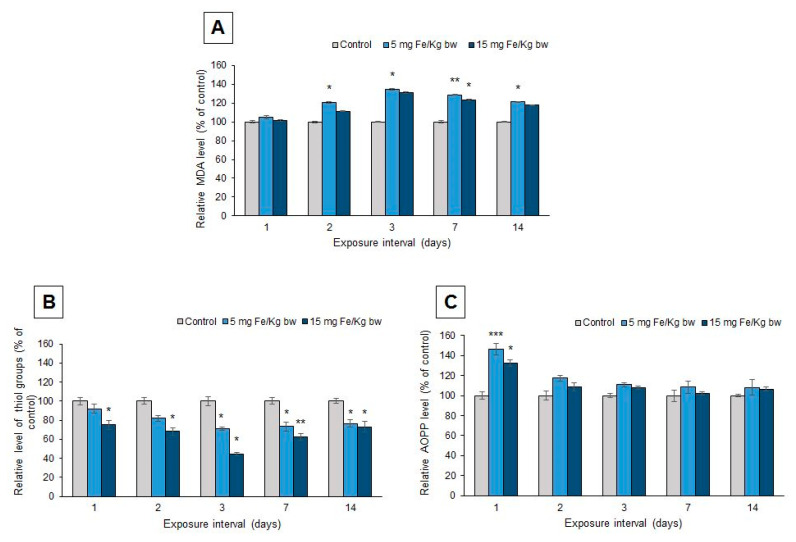
Effect of MNP-DSPE-PEG in the liver of CD1 mice on (**A**) malondialdehyde (MDA), (**B**) protein thiol groups (P-SH), and (**C**) advanced oxidation protein products (AOPP) levels up to 14 d. The results were expressed as mean ± standard deviation (SD) and represented as percentages related to the control group; the data were considered statistically significant when * *p* < 0.05; ** *p* < 0.01; *** *p* < 0.001 versus the control group.

The level of lipid peroxidation was appreciated by measuring the amount of MDA products generated up to 14 d after injection (Figure 8). Thus, the results showed a significant elevation of the MDA level starting with the second day and maintained until the end of the experiment. The maximum increase was observed after 3 d by 35% and, respectively, 31% for the two conditions (5 and 15 mg Fe/kg bw). After 7 d, a slight decrease was observed, but the MDA level remained over the one found in the control group.

Protein thiol groups (P-SH, Figure 8B) and advanced oxidation protein products (AOPP, Figure 8C) were analyzed as markers for protein oxidations. The injection of MNP-DSPE-PEG caused a dose-dependent decrease in the P-SH level in the mice liver. The maximum decrease was observed after three days by 29% in mice injected with 5 mg Fe/kg bw and by 56% in those injected with 15 mg Fe/kg bw, compared with the control group. On the other hand, the AOPP level increased after the first day of exposure by 46% for the low dose and, respectively, by 32% for the high dose, compared to the control. After this interval, the level of AOPP changed and no significant increase was registered up to 14 d.

## 4. Discussions

This paper presents the biological properties and imaging efficacy of a new type of contrast agent, represented by magnetite nanoparticles encapsulated in a modified phospholipid micelle layer exhibited at the hepatic level in CD1 mice. Studies regarding the toxicity of these nanoparticles in other organs such as spleen [61] and lungs [51] were previously presented. The current work presents the status of the hepatic oxidative stress markers following the administration of nanoparticles to CD1 mice, their biodistribution, accumulation, and elimination, as well as the imaging properties at the hepatic level during two weeks.

Liver and kidneys maintain the homeostasis of organisms, by buffering some pH changes limited in amplitude and by maintaining the ionic power and the chemical composition of blood plasma [62]. Thus, establishing an exact path of the nanoparticles route in the body could be difficult considering also the relationship between organs and the influence of external factors. Under certain conditions, fractions of nanoparticles could be retained in the body for long periods due to incomplete excretion and, consequently, could disrupt the normal functioning of organs and tissues by inducing toxicity in organs such as liver, spleen, lymph nodes, and lungs [63].

The variation of hepatic markers analyzed from mice blood serum was in accordance with biochemical data from liver tissue. Slight modifications of liver enzymes (AST and ALT) activities were observed on short term (3 d) in blood serum after nanoparticle administration but were not statistically significant indicating at most temporary liver damage most likely due to the iron overload. Similarly, no significant changes in GGT activity were noticed in the exposed mice blood in the first 3 d meaning that no severe hepatic injury was produced. After 7 d of exposure, the enzymatic activities in blood serum were restored.

Cellular uptake of iron oxide nanoparticles includes clathrin and caveolin dependent and independent pathways as well as macropinocytosis [64,65]. The Prussian Blue staining revealed the accumulation of nanoparticles in liver tissue after two- and three-days postadministration for both doses, more pronounced for the higher dose. After 7 d, the MNP-DSPE-PEG were no longer visible in the hepatic parenchyma.

Furthermore, the NMRD profile revealed the magnetic properties of magnetite nanoparticles used in this study. Considering that proton relaxation mainly occurs at the interface between MNP and its aqueous environment, [66] the coating largely influences the relaxation process. The relaxivities are affected by the surface properties of MNP, as well as by their chemical composition and size [67]. The presence of DSPE-PEG coating is expected to enhance the hydration state of the micelle and to limit concomitantly the exchange of water molecules between the surface of MNP and their environment, which is reflected by the r_2_ enhancement and r_1_ decline, with a consequent elevation of r_2_/r_1_ ratio. The clustering of MNP in phospholipid enclosed compartments such as cells, liposomes, and micelles are furthermore known to contribute to the striking r_2_ enhancement. The restrained diffusion of water molecules across the phospholipid barrier is moreover responsible for r_1_ drop due to the absence of direct contact with the magnetic core [68]. Similar decreases of r_1_ at higher magnetic fields for SPIONs were noticed previously [69,70]. The obtained relaxivity values indicated that these MNP have a much larger r_2_ compared to r_1_ and, therefore, they could be used as negative contrast agents in MRI studies, according to our findings. Other previous studies have shown that MNP-based contrast agents, such as the commercial product Resovist, as well as other compounds such as polyglycerol-grafted MNP, applied by intravenous injection, are rapidly taken by the liver in 6–10 min after administration. In these cases, the negative contrast persisted in the liver for about one and a half hours. Over time, the contrast has become weaker, probably due to the coating (that made it susceptible to renal excretion) [71]. Zhu et al. observed a significant negative contrast in the rat liver area 10 min after intravenous injection of colloidal magnetite nanoparticles embedded in poly (St-NIPAM) [72]. The negative contrast was maximal at one hour after the administration of the compound and persisted for up to 8 h. Imaging studies showed the change of the MRI signal intensity after the administration of MNP-DSPE-PEG in the liver starting with the first 41 min after injection of the nanoparticle suspension. The darkening of areas of interest on the T_2_-weighted images by decreasing the MRI signal was proportional to the accumulation of iron and an increase of the relaxation rates R_2_ in the liver. 

Yan et al. confirmed the ability of magnetite nanoparticles encapsulated in polymeric micelles (composed of poly amphiphilic copolymers (HFMA-co-VBK)-g-PEG) to produce a significant negative contrast of liver 30 min after their injection, the high contrast persistence up to 4 h [73]. The same observation was made by Ahmad et al. on MNP encapsulated in silicon. The hepatic area of the axial T_2_-weighted images was pronounced and visibly darker in post than in precontrast images [74].

Internalized MNP are probably degraded into iron ions in lysosomes under the influence of hydrolysis enzymes that act at a low pH [75]. An accumulation of free iron ions in the cytoplasm of cells could lead to an excessive ROS generation.

In mammals, the liver has developed a sophisticated antioxidant defense (enzymatic and nonenzymatic) to maintain the redox homeostasis. When ROS are overproduced, this state is disturbed, resulting in oxidative stress, which plays a critical role in liver diseases. Besides, the alterations of lipids and proteins that result after oxidative stress installation, this state could modulate pathways that regulate protein expression, gene transcription, cell apoptosis, and hepatic stellate cell activation. Thus, oxidative stress is considered as one of the main pathological mechanisms that result in the initiation and progression of liver diseases, such as nonalcoholic steatohepatitis chronic viral hepatitis and alcoholic liver diseases [32]. A recent study showed that Fe_3_O_4_ nanoparticles possess intrinsic enzyme-like activity catalyzing the decomposition of H_2_O_2_ to water and oxygen, resembling catalase [9]. Thus, this intrinsic activity of Fe_3_O_4_ nanoparticles may act to antagonize the accumulation of toxic ROS induced by themselves, and thereby to modulate the extent of cellular oxidative stress.

Our investigation showed that the specific activity of CAT in the liver increased in the first two days after nanoparticle administration probably as an antioxidant defense response but decreased on the third day, most likely as a consequence of the increase of iron ions level, which are inhibitors of the CAT activity [76]. Similarly, an increase of the total SOD activity (Cu/Zn-SOD and Mn-SOD) was observed in the first 2 d of the experiment that could be a result of the superoxide anion generation in the liver formed by the activation of NADPH oxidases in phagocytic and nonphagocytic cells due to their physical interaction with nanoparticles. On the third day, SOD activity dropped possibly due to the iron accumulation that decreased on one hand the activity of Cu/Zn-SOD due to its negative effect on the copper ion state [77] and the other, due to its influence on the redox potential of the active catalytic center of Mn-SOD to a level that is incompatible with the dismutation reaction of superoxide anions [78]. The GPx activity was decreased probably due to the fact that hydrogen peroxide level generated by Fenton reaction was high during the first three days. In this situation, CAT is more effective than GPx due to its K_M_ value. At the same time, the variation of GST activity was increased due to its action in detoxification reactions of electrophilic substances such as malondialdehyde during the entire time interval.

The G6PDH and GR enzymes play an essential role in maintaining glutathione in its reduced form [79]. According to our data, the activity of these enzymes in liver tissue was significantly higher compared to control after the first day of exposure to nanoparticles but was restored to the end of the experiment. However, we found a diminution of GSH content in the exposed mice compared to control mice at all exposure intervals, probably as a consequence of the oxidative stress generated by the accumulation of iron ions and its use as a substrate in the reaction catalyzed by GST. These effects are similar to those noticed in human Chang hepatocytes exposed to silver nanoparticles [80]. In contrast with our study, it was shown that the gold nanoparticles induced no significant changes in the level of this tripeptide in rat liver [81].

ROS might attack in a first step polyunsaturated fatty acids from biological membranes, generating a lipid peroxidation cascade, which results in the formation of MDA-like aldehydes [82]. Previous studies have shown that MDA levels increased in a dose-dependent manner in the liver and kidneys of Kunming mice intraperitoneally injected with MNP [83]. In our study, the MDA level did not increase on the first day most likely due to the effective activity of GST, but this did not decrease completely the MDA concentration to the normal level after 3, 7, and 14 d of exposure. 

Jain and colleagues tested OA-Pluronic (a coating consisting of oleic and Pluronic acid) coated MNP in Dawley rats at a dose of 10 mg Fe/kg bw, injected intravenously, and did not find significant changes in liver enzymes activities [84]. The level of lipid hydroperoxides increased slightly at the liver level, returning to normal after one week. After 6 h postadministration, 55% of the amount of injected iron accumulated in the liver, and after one day this amount was reduced up to 20%. Moreover, it was shown that these nanoparticles did not affect cell integrity or tissue morphology [85]. Although the accumulation of nanoparticles was quantitatively higher in the liver, the level of lipid hydroperoxides did not increase directly proportionally with iron accumulation, and this might be due to the individual ability of each organ to counteract the effects caused by MNP [85]. On the other hand, another study reported that following iron overload, both levels of liver iron and lipid peroxidation increased in a dose-dependent manner in mice. The responsible for this fact could be ROS resulting from the release of iron ions from lysosomes, which can no longer be stored in ferritin and metallothioneins [63]. The degree of protein oxidations was reflected by AOPP and protein thiol groups levels. In our case, an increase of AOPP was observed only on the first day of exposure. The protein thiol groups level presented a similar profile to that of GSH, which could suggest that they cooperate in counteracting ROS generated by exposure to MNP covered with DSPE-PEG micelles. These data suggest that only moderate oxidative stress was installed postinjection of MNP-DSPE-PEG in the mice liver that is unable to produce severe injury to the tissue. According to the findings of Ma et al., [83], injection of a dose over 40 mg/Kg bw of Fe_3_O_4_ nanoparticles can induce significant alterations including DNA damage in the mice liver.

From the histopathological analysis as well as from the biochemical data, MRI and relaxometry, it appears that after intravenous administration, MNP encapsulated in phospholipid polymeric micelles were progressively translocated into the liver. The most significant biochemical changes occurred in the first 3 d after exposure when the most severe histopathological and immunohistochemical changes were observed. The level of the majority of the analyzed oxidative stress markers was changed transiently, returning close to the control level at the end of the experimental period but the levels of some of them such GSH, protein thiols groups, and MDA were significantly altered up to 14 d. However, the maxim alterations were recorded also after 3 d postinjection after which a tendency of recovery of their levels occurred. Taking into account all the data obtained during the two weeks of exposure, we can conclude that the antioxidant defense system of the liver counteracted efficiently the oxidative stress induced by the exposure to this type of nanoparticles. 

In summary, the results of our study indicated that MNP encapsulated in DSPE-PEG micelles might be potential candidates for use as contrast agents, as they present a strong contrast on the T_2_-weighted images up to 3 h in the liver tissue and exert low toxicity which liver defense systems can counteract. However, more detailed investigations are needed to translate their use in clinical applications (dose optimization, broadening the area of investigation, determining effects on other organs and correlation of all data, determination of possible elimination routes, analysis of signaling pathways).

## Figures and Tables

**Table 1:** The values of MNP-DSPE-PEG relaxivities at 20 and 60 MHz, 37 °C.

Parameters	r_1_ (s^−1^ mM^−1^)	r_2_ (s^−1^ mM^−1^)	r_2_/r_1_ (s^−1^ mM^−1^)
**20 MHz**	15.39	151.23	9.83
**60 MHz**	3.85	183.48	47.66
